# P60 Morphological criteria of tuberculous inflammation in the complex evaluation of chemotherapy effectiveness in pulmonary TB

**DOI:** 10.1093/jacamr/dlaf230.067

**Published:** 2025-12-04

**Authors:** E M Petrunina, S N Skornyakov

**Affiliations:** National Medical Research Center for Phthisiopulmonology and Infectious Diseases, Yekaterinburg Branch, Yekaterinburg, Russia; National Medical Research Center for Phthisiopulmonology and Infectious Diseases, Yekaterinburg Branch, Yekaterinburg, Russia

## Abstract

**Background:**

The prevalence of *Mycobacterium tuberculosis* (MTB) strains resistant to anti-TB drugs (ATD) remains a major challenge for TB treatment. Surgically obtained lung material plays an important role in assessing both the pathogen’s drug resistance and the host immune response. The relationship between molecular DR markers, the morphological activity of inflammation and treatment outcomes warrants further investigation.

**Objectives:**

To evaluate microbiological and histological characteristics of pulmonary resection specimens from patients receiving standardized anti-TB chemotherapy, and to determine correlations between treatment efficacy, morphological markers of inflammatory activity and presence of drug resistance-associated mutations in the MTB genome.

**Materials and methods:**

A cohort of 150 adult patients underwent lung resection in 2024 for localized TB. Patients with HIV or significant comorbidities were excluded. Indications for elective surgery, selection of treatment regimens and evaluation of their effectiveness were conducted in accordance with national Clinical Guidelines. MTB isolates from resected lung tissue were tested for phenotypic drug susceptibility and screened for resistance mutations to isoniazid (H), rifampicin (R) and fluoroquinolones (Fq). Histological activity of inflammation was graded as active (predominant exudative-necrotic inflammation), moderately active (intermediate patterns), or low (fibrosis predominance with minimal inflammatory response). Correlation between treatment effectiveness and morphological activity was assessed using Kendall’s τ (α=0.05).

**Results:**

Viable MTB were cultured from 20 of 150 patients; 17 showed active and 3 moderately active inflammation. In all these patients, H+R resistance was found, combined with DR to a wide range of reserve ADT, while only 5 (25%) patients registered a match in the structure of drug resistance to the set of drugs used before surgery. MTB DNA was detected in all specimens. Resistance mutations were identified in 23 patients for H, in 5 for R, in 60 for H+R and in 17 for combined H+R+Fq resistance. Inflammatory activity was highest in patients with H+R+Fq resistance (78% with high activity), intermediate with H+R (50% high) and mostly moderate in cases of monoresistance. When therapy regimens were adequately matched to resistance profiles, no significant association between mutations and inflammation was observed. A significant negative correlation (τ=–0.76, critical T=0.51) was found between chemotherapy effectiveness and inflammation activity. Among 41 patients whose treatment regimen corresponded to molecular data, 61% exhibited moderate and 39% low inflammation. Among patients with regimens inconsistent with molecular findings, 52% showed high activity, 33% moderate and only 15% low inflammatory activity. Resistance markers most frequently in combination with active inflammatory process were detected in patients who received preoperative treatment regimens for drug-susceptible TB due to the lack of baseline data on DR of the pathogen (in 4 out of 5, 80%).

**Discussion:**

Direct comparison of detailed histological grading of tuberculous inflammation with molecular resistance profiles and chemotherapy outcomes underscores the importance of incorporating morphological criteria in the assessment of treatment outcomes.

**Conclusions:**

Histological features of active inflammation are a critical indicator of insufficient chemotherapy effectiveness and the necessity for treatment modification. The presence of resistance mutations, provided that ADT is correctly selected, does not significantly affect the severity of histological signs of inflammation.

